# Mathematical Modeling and Models for Optimal Decision-Making in Health Care

**DOI:** 10.1155/2019/2945021

**Published:** 2019-08-14

**Authors:** Giedrius Vanagas, Tomas Krilavičius, Ka Lok Man

**Affiliations:** ^1^Institute of Pharmacoeconomics, LT-51052 Kaunas, Lithuania; ^2^Vytautas Magnus University, Kaunas, Lithuania; ^3^Lithuania and Baltic Institute of Advanced Technology, Vilnius, Lithuania; ^4^Xi'an Jiaotong-Liverpool University, Suzhou 215123, China; ^5^Swinburne University of Technology Sarawak, Kuching, Malaysia

Many aspects in the management of healthcare systems are quantitative, the amount of data within the health care increases by the minute, and, in reality, it makes difficult for healthcare systems to identify the insights into what is most valuable for the patients. Data-driven approach to health (or health economic) outcome assessment, artificial intelligence, and mathematical, computational, methodological, and technological advances are the core of effective healthcare system management [1–3].

Modeling in medicine is a valuable tool in the planning and evaluation of interventions, especially when a clinical trial is ethically or logistically impossible [4, 5]. The development of such mathematical models used to simulate medical outcomes is a growing area in medicine. The mathematical modeling is known by various names like predictive modeling, simulation, or decision analysis. In general, modeling techniques are used for health service planning, effectiveness and outcome assessment, healthcare financing and budget impact assessment, health economic assessments, infectious disease surveillance, health service outcomes predicting purposes, and other applications in health care. Mathematical modeling is also helpful when limitations like a rare event prohibit implementing RCT and similar studies or expanding research on actual patients due to time, ethical, legal, financial, technical, and other limitations [6, 7].

With this special issue, we add to the literature by providing case studies and practical examples of mathematical modeling and models for optimal decision-making in health care. We aim to address questions from data analytics, solving problems in predicting outcomes for clinical medicine and public health.

Blood pressure (BP) is one of the indispensable elements of physiological health characteristics and a significant indicator for predicting and diagnosing hypertension and cardiovascular diseases. Q. Wang et al. proposed a two-domain fusion model to estimate BP continuously from pulse wave acquired with a pressure sensor. In more detail, the optimal external pressure applied on the pressure sensor was first determined in order to capture pulse wave in the radial artery. The captured pulse wave was then processed in both the time and frequency domains via filtering and fast Fourier transform. A set of features were extracted from these two domains and input into a neural network along with blood pressure values measured by a commercial sphygmomanometer for training. Finally, the model was tested on new data for accuracy evaluation, and the proposed two-domain fusion method achieved a high degree of accuracy in measuring blood pressure.

H. Shang et al. proposed an improvement for ECG analysis, namely, improved sliding window area method for *T* wave detection. It allows better detection of *T* wave onset and offset, which allows improving clinical diagnosis as well as daily heart monitoring.

A. K. Heikhmakhtiar and K. M. Lim proposed computational prediction of the combined effect of CRT and LVAD on cardiac electromechanical delay in the failing ventricle with left bundle branch blocked (LBBB) and right bundle branch blocked (RBBB) conditions. The subjects were normal sinus rhythm, LBBB, RBBB, LBBB with CRT-only, RBBB with CRT-only, LBBB with CRT + LVAD, and RBBB with CRT + LVAD. The results showed that the CRT-only shortened the total electrical activation time (EAT) in the LBBB and RBBB conditions by 20.2% and 17.1%, respectively. The CRT-only reduced the total mechanical activation time (MAT) and electromechanical delay (EMD) of the ventricle under LBBB by 21.3% and 10.1%, respectively. Furthermore, the CRT-only reduced the contractile adenosine triphosphate (ATP) consumption by 5%, increased left ventricular (LV) pressure by 6%, and enhanced cardiac output (CO) by 0.2 L/min under LBBB condition. However, CRT-only barely affected the ventricle under RBBB condition. Under the LBBB condition, CRT + LVAD increased LV pressure and CO by 10.5% and 0.9 L/min, respectively. CRT + LVAD reduced ATP consumption by 15%, shortened the MAT by 23.4%, and shortened the EMD by 15.2%. In conclusion, they computationally predicted and quantified that the CRT + LVAD implementation is superior to CRT-only implementation particularly in HF with LBBB condition.

Magnetic resonance imaging has been widely used in diagnostic imaging for general checkup in clinical practice, especially in detection and diagnosis of brain diseases. However, brain MR imaging has some lacks such as noise, intensity inhomogeneity, low contrast, and partial volume effect , which brings serious obstacles to segment the brain MR images. The study of J. Song and Z. Zhang presented a novel and more robust method to noise in the brain magnetic resonance imaging, together with a more effective estimation method of the bias field.

Automatic segmentation of different images is one of the most important topics in medicine. L. Cao et al. discuss application of Random Forests Stacks for automatic segmentation of pathological glomerular basement membranes in TEM images. It allows faster observation of morphological changes, reducing manual and laborious work of specialists. Another exercise in image processing is discussed in J. Song and Z. Zhang. Improvements for brain tissue segmentation and bias field correction of MR images are proposed and evaluated. The results are promising and potentially can deal with noise in brain MR images.

In conventional radiotracer and drug development, poor bench-to-bedside translation often results due to the differences between in vitro and in vivo conditions [8]. The study by Y.-H. Nai and H. Watabe evaluated the feasibility of extending the amyloid-validated screening methodology to support the development of tau PET radiotracers, where more challenges like off-target binding exist. This is the first in silico method investigated, which uses the physicochemical and pharmacological properties of the compounds to support tau PET radiotracers developments. 22 PET radiotracers reported to bind to tau proteins were investigated, including 9 clinically applied and tau-focused radiotracers. The study supported the use of the screening methodology in radiotracer development by allowing comparison of candidate radiotracers with clinically applied radiotracers based on SUVR, with respect to binding to a single target, and provides some insights to guide the development of in silico models in supporting the development of tau radiotracers.

Automatic identification of relevant biomarkers is one of the important steps towards personalized treatment. B. Haller et al. evaluate applicability of a number of methods, for example, Cox regression with linear interaction, Multivariable Fractional Polynomials for Interaction (MFPI), Local Partial Likelihood Bootstrap (LPLB), and the Subpopulation Treatment Effect Pattern Plot (STEPP), for biomarker identification. Experiments on randomized clinical trials show that the Cox regression works best when interactions are monotonous and the number of events is low. When complexity increases, MFPI and LPLB outperform other methods. The authors recommend application of statistical methods developed for assessment of interactions between continuous biomarkers and treatment instead of arbitrary or data-driven categorization of continuous covariates.

The study of D. Liu et al. applies similarity measures of single and interval valued neutrosophic sets based on Euclidean distance for diagnostics. Novel theoretical model is developed in the paper, and its effectiveness is demonstrated on generalized diagnosis, showing that it performs well in solving a multiple criteria decision process. The proposed similarity measures were applied to medical diagnosis decision problems, and a number of examples were used to illustrate the feasibility and effectiveness of the proposed similarity measure.

Coreference resolution is a challenging part of natural language processing (NLP) with applications in machine translation, semantic search, and other information retrieval and decision support systems. V. Žitkus et al. presented a method for coreference resolution in the Lithuanian language and its application for processing e-health records from a hospital reception. The novelty of their proposed method is the ability to process coreferences with minimal linguistic resources, which is important in linguistic applications for rare and endangered languages. Their experimental results have shown that coreference resolution is applicable to the development of NLP-powered online healthcare services in Lithuania.

Computer-aided models for mammographic breast cancer diagnosis (MBCD) have been explored for over thirty years [9]. The study of L. Zou et al. dedicated to the technique of CNN applied in a specific application of MBCD, and it aims to provide clues on how to use CNN in intelligent diagnosis. This study is restricted to peer-reviewed journal publications, and consequently, technical details and pros and cons of each model can be delivered. Furthermore, based on how to use CNN techniques, the MBCD models are broadly categorized into three groups. One is to design shallow models or to modify existing models for decreased time cost and medical instances for training; another is to make the best use of a pretrained CNN model by transfer learning and parameter fine-tuning; and the third is to take advantage of CNN models for feature extraction, while the differentiation between malignant and benign lesions is based on machine learning classifiers. At last, findings, challenges, and limitations are summarized, and some clues on the future work are also given. At present, the design and use of CNN-based MBCD is at its early stage and result-oriented. The ultimate goal of using deep learning tools is to facilitate clinical practice. This review provides benefits to scientific researchers, industrial engineers, and those who are devoted to intelligent cancer diagnosis.

The past application of mathematical models in medicine also has been proven useful in cardiovascular diseases (CVDs). The study of O. Saidi et al. aimed to describe a comprehensive Markov model based on both a probabilistic multivariate approach and simple linear regression metamodeling using the model to evaluate the effects of increases in uptake of stroke treatments, lifestyle changes, and primary prevention among the Tunisian population aged 35–94 years in 2025. It examined three interventions: improved medical treatments in the acute phase, secondary prevention of stroke by increasing the prescribing of statins, and primary prevention aiming to reduce salt intake.

Type-1 diabetes is a condition caused by the lack of insulin hormone, which leads to an excessive increase in blood glucose level. The glucose kinetics process is difficult to control due to its complex and nonlinear nature and with state variables that are difficult to measure. P. D. Ngo et al. proposed a method for automatically calculating the basal and bolus insulin doses for patients with type-1 diabetes using reinforcement learning with a feedforward controller. The proposed controller also improved the blood glucose responses and prevented hypoglycemia condition. Simulation of the control system in different uncertain conditions provided insights on how the inaccuracies of carbohydrate counting and meal-time reporting affect the performance of the control system. As a conclusion, the proposed controller is an effective tool for reducing postmeal blood glucose rise and for countering the effects of external known events such as meal intake and maintaining blood glucose at a healthy level under uncertainties.

In the paper of B. H. Lichae et al., the fractional-order differential model of HIV-1 infection of CD4+ T-cells with the effect of drug therapy has been introduced. There are three components: uninfected CD4+ T-cells, *x*, infected CD4+ T-cells, *y*, and density of virions in plasma, *z*. The aim is to gain numerical solution of this fractional-order HIV-1 model by the Laplace Adomian decomposition method (LADM). The solution of the proposed model has been achieved in a series form. Moreover, to illustrate the ability and efficiency of the proposed approach, the solution has been compared with the solutions of some other numerical methods. The Caputo sense has been used for fractional derivatives.

Beds are key, scarce medical resources in hospitals. The study of L. Luo et al. aimed to balance the utilization of existing beds in a large tertiary hospital in China. The author developed a data-driven hybrid three-stage framework incorporating data analysis, simulation, and mixed integer programming to minimize the gaps in bed occupancy rates (BOR) among different departments. The first stage is to calculate the length of stay (LOS) and BOR of each department and identify the departments that need to allocate beds. In the second stage were used a fitted arrival distribution and median LOS as the input to a generic simulation model. In the third stage was built a mixed integer programming model using the results obtained in the first two stages to generate the optimal bed allocation strategy for different departments. The case study demonstrated the effectiveness of the proposed data-driven hybrid three-stage framework and provides hospital bed policy makers with a feasible solution for bed allocation.

Mathematical models are often used and prove their applicability for optimal decision-making. They are also useful to derive estimates of rare or future events from recorded intermediate points. When developing models, decisions are needed about the appropriate level of complexity to be represented and about model structure and assumptions.
